# Towards a Triple Helix based efficiency index of innovation systems

**DOI:** 10.1007/s11192-022-04304-x

**Published:** 2022-04-09

**Authors:** Milica Jovanović, Gordana Savić, Yuzhuo Cai, Maja Levi-Jakšić

**Affiliations:** 1 Faculty of Organizational Sciences, University of Belgrade, Jove Ilića 154, Belgrade, Serbia; 2 Faculty of Management and Business, Tampere University, Kalevantie 4, Tampere, Finland

**Keywords:** Triple Helix, Data envelopment analysis, European innovation paradox, Efficiency, Performance measures, OECD, O11, O38, C44, 91B99, 90B99

## Abstract

This article presents a novel application of a two-phase Data Envelopment Analysis (DEA) for evaluating the efficiency of innovation systems based on the Triple Helix neo-evolutionary model. The authors identify a niche to measure Triple Helix-based efficiency of innovation systems scrutinizing different methodologies for measuring Triple Helix performance and indicating different perspectives on policy implications. The paper presents a new Triple Helix-based index that engages a comprehensive dataset and helps provide useful feedback to policymakers. It is based on a set of 19 indicators collected from the official reports of 34 OECD countries and applied in a two-phase DEA model: the indicators are aggregated into pillars according to the Assurance Region Global and DEA super-efficiency model; pillar scores are aggregated according to the Benefit-of-the-Doubt based DEA model. The results provide a rank of 34 countries outlining strengths and weaknesses of each observed innovation system. The research implies a variable set of weights to be a major advantage of DEA allowing less developed countries to excel in evaluating innovation systems efficiency. The results of Triple Helix efficiency index measurement presented in this paper help better account for the European Innovation Paradox.

## Introduction

The COVID-19 pandemic shows the importance of synergistic effects of multiple stakeholders’ joint efforts in overcoming threats and reducing the risks of a global crisis which leads to disease, death, economic decline, business failure and job losses. Effective collaboration between government, industry and academia is crucial for economic survival, where a crisis may be overcome using innovative solutions (Layos & Peña, 2020; Niankara et al., 2020). It is argued that in regular circumstances, collaboration and cross-sectoral joint efforts are equally effective and substantially contribute to the prosperity and sustainability of an innovative society (Lerman et al., 2021).

The Triple Helix model, originated by Etzkowitz and Leydesdorff (1995), is widely acknowledged as a conceptual tool that promotes innovation and entrepreneurship through better understanding, cooperation and interaction between university, industry, and government institutions, and supports economic growth and innovation policy design in turn (Cai & Liu, 2020; Galvao et al., 2019). The Triple Helix is effective at both the national and regional levels (Leydesdorff & Deakin, 2011; Rodrigues & Melo, 2012), while its cross-sectoral interactions are recognized as a key force of circular economy and sustainability (Anttonen et al., 2018; Scalia et al., 2018; Ye, & Wang, 2019). In addition to social benefits, the usefulness of Triple Helix interactions has also been demonstrated organizationally. Hernández-Trasobares and Murillo-Luna (2020) have confirmed that the cooperation of industry, academia, and government in R&D contributes to success in business innovation.

National efforts to enhance innovation policies and competitiveness (e.g., Smart Specialization Strategy by EC JRC, EC HEG KET) point to a paradox of increased innovation investments not fulfilling the proclaimed sustainable development goals, designated as the European Innovation Paradox (Dosi, Llerna, & Labini, 2006). Some recent studies have attempted to find methods and tools for overcoming this Paradox, focusing on the assessment innovation systems indicators. Their conclusions highlighted the changes that needed to be made accordingly, based on the approaches and methodology used for innovation systems evaluation (Argyropoulou et al., 2019). Argyropoulou et al. recognize that the Triple Helix model is a tool to be adapted for wider application in overcoming the effects of the European Innovation Paradox, aimed at reaching a “harmony for knowledge-based economy” (Argyropoulou, Soderquist, & Ioannou, 2019).

While the development of the Triple Helix model needs to be pre-structured and coordinated (Cai & Etzkowitz, 2020), the interplay of the Triple Helix actors is difficult to manage as it requires activities of multiple, disparate sectors within complex subsystems (Jovanović et al., 2020; Ranga & Etzkowitz, 2013). New approaches to the theory and practice of the Triple Helix concept are constantly arising (see e.g., Todeva et al., 2019), but challenges remain on how to best manage Triple Helix interactions in order to improve the effectiveness and efficiency of the innovation ecosystem. Successful Triple Helix dynamics requires us to measure and evaluate the performance of Triple Helix actors (Dankbaar, 2019; Sá et al., 2019). For this purpose, a range of measurements have been developed (e.g., Jovanović et al., 2020; Leydesdorff, 2003; Leydesdorff & Ivanova, 2016; Mêgnigbêto, 2018; Priego, 2003; Xu & Liu, 2017). Measuring Triple Helix performance helps identify both good practices and possible deficiencies in interactions, enables comparison between countries and regions, solicits solutions to challenges, and points to strategies taking advantage of opportunities.

Cirilloa et al. (2018) emphasize that the origins of the European Paradox may be better examined when using a proper set of measurements and scientific indicators. Extensive literature review has helped identify three major research gaps. Firstly, although literature on quantitative measurement of Triple Helix abounds, there are still very few studies which compare Triple Helix performance measurement tools. Secondly, the existing approaches are highly focused and lack the capacity and specific design to detect strengths and weaknesses of systems based on the measurements, consequently failing to contribute to innovation strategy and policy development. Finally, the European Innovation Paradox has been insufficiently examined from the Triple Helix perspective, which is recognized as having the strength to shed more light on causal relationships within this phenomenon.

To bridge the above stated literature gaps, this paper poses two research questions: (1) How may the existing approaches of measuring Triple Helix be assessed? and (2) How can Triple Helix-based efficiency at a national level be measured using a comprehensive set of indicators that provide understandable and useful feedback to policymakers on system improvement? The first research question is tackled by analyzing different research studies on measuring Triple Helix. This helped evaluate the observed aspects of Triple Helix interactions and identify a niche for further research. As for the second research question, we have developed a novel application of the Data Envelopment Analysis (DEA) to measuring Triple Helix-based efficiency scores of innovation systems. Empirically, 34 OECD countries are compared according to their efficiency in transforming inputs of an innovation system into innovative outputs fostered by Triple Helix actors. The results of this measurement should provide useful feedback on innovation action for policymakers, particularly as a potential solution for the European Innovation Paradox.

The paper is structured as follows: Sect. 2 provides literature review on the existing approaches to measuring Triple Helix performance and Data Envelopment Analysis. Section 3 introduces the development of the proposed index of Triple Helix-based efficiency. Section 4 presents the results of measuring the Triple Helix efficiencies of 34 OECD countries. Section 5 discusses the implications of our findings for existing and potential stakeholders. Finally, we conclude by indicating the limitations of our study, and suggesting the direction for further research.

## 2. Literature review: Triple Helix concept and measures

The concept of the Triple Helix model originated in 1995 to help comprehend the dynamics of interactions between university, industry, and government, which in turn would promote innovation and entrepreneurship (Etzkowitz & Leydesdorff, 1995). Since then, it has become prominent in scholarly research and policy discourse. An area of Triple Helix studies has focused on measuring Triple Helix performance. Literature review in this paper is mostly based on studies related to Triple Helix measurement found in prominent scientific databases (Web of Science, Scopus, and Google Scholar) under the key words ‘Triple Helix indicators’, ‘Triple Helix measures’, ‘Triple Helix measuring’, ‘Triple Helix performance’ and ‘Triple Helix evaluation’. This section aims to clarify the state-of-the-art of research in the field and to identify existing research gaps, rather than provide a systematic literature review report.

### Why is measuring the Triple Helix important?

Cross-sectoral collaborations are key to successful innovation, since joint forces and efforts allow for a better understanding of diverse perspectives (Singer & Oberman Peterka, 2012), facilitate knowledge exchange and distribution, provide additional opportunities (e.g., in funding, projects, products), reduce knowledge redundancy (Leydesdorff & Ivanova, 2016), and boost innovative and economic performance (Luengo & Obeso, 2013; Razak & White, 2015). The Triple Helix model is aimed at better understanding of complex interactions among multiple university, industry and government actors which may foster innovation and entrepreneurship (Etzkowitz & Leydesdorff, 2000). Recognizing the usefulness of this model, governments and decision-making bodies have strived to not only design policies which would improve their innovation systems, but also allocate resources to promote Triple Helix interactions (Cai & Etzkowitz, 2020). However, such policies may only be effective when informed by a purely evidence-based Triple Helix model, e.g., through measuring Triple Helix synergies (Leydesdorff & Smith, 2021). Park and Leydesdorff (2010) come to important conclusions using the so-called Triple Helix indicator to evaluate the effectiveness of governmental policies in South Korea and their impact on co-authorship collaboration patterns.

Three major arguments are put forward concerning the importance of Triple Helix measurement. First, it provides a control mechanism for policy implementation which helps estimate its efficiency and effectiveness (e.g., Brignall & Modell, 2000; Ivanova & Leydesdorff, 2015). Secondly, performance evaluation is essential to improve Triple Helix interactions as it permits the detection of weak links and good practices within the Triple Helix systems observed (e.g., Keramatfar & Esparaein, 2014; Lebas, 1995). Finally, measuring the Triple Helix efficiency may be used in developing ranking tools for innovation competitiveness on a global scale (e.g., Jovanović et al., 2020; Ye & Wang, 2019).

### Evaluating Triple Helix performance – current methodological approaches

The following table presents a summary of approaches to measuring Triple Helix performance (Table 1).

**Table 1:** Existing measures of Triple Helix performance

What is measured:	References	Triple Helix actors evaluated	Pros	Cons
Patent activity	OECD (2020a, b), Meyer et al. (2003)	All three actors	Important measure of R&D performance	Difficulties in the patenting process Does not evaluate overall Triple Helix performance
Bibliometrics & publishing activity	Villanueva-Felez et al. (2013); Xu et al. (2015); Priego (2006)	University, Industry	Offers insight into R&D cooperation of industry and academia	Evaluates only output measures. Does not evaluate government performance Does not evaluate overall Triple Helix performance
Academic spin-offs	Lawton Smith and Ho (2006); Fini et al. (2017); Samo& Huda (2019)	University	Indicates the level of entrepreneurial orientation of universities within a selected country	Limited data on the number of spin-offs within a country Does not evaluate overall Triple Helix performance
Mutual information	Leydesdorff (2003); Leydesdorff et al. (2006); Leydesdorff& Fritsch (2006); Leydesdorff& Sun (2009); etc	All three actors	Evaluates synergy strength and interactions within a system	Based solely on bibliometric analysis. Does not evaluate any other type of interaction
Interrelations	Villanueva-Felez et al. (2013)	All three actors	Evaluates strength of collaboration between academia and non-academic environment	Focused only on social networks. Neglects other important aspects of the Triple Helix model
Multivariate approaches	Tijssen (2006); Tarnawska and Mavroeidis (2015); Marinković et al. (2016); Egorov and Pospelova (2019); Ivanova et al. (2019); Jovanović et al. (2020)	All three actors	Combines disparate aspects of Triple Helix performance	Effectiveness depends on the aggregation methods selected Sensitive to the selection of indicators Missing data within some systems

### Identified gaps in Triple Helix performance measurements

Extensive literature review has suggested a niche for further development of a Triple Helix-based index for measuring comprehensive performance. While current measurements are mainly based on a single indicator or multiple measure reports, some available comprehensive datasets have not been fully utilized for measuring Triple Helix performance due to methodological challenges. For instance, the OECD Science, Technology and Innovation (STI) Outlook provides a comprehensive overview of major trends in STI development of OCED countries and may assist policymakers in detecting global patterns and help define and update their STI strategies accordingly (OECD, 2020a). With a set of almost 130 indicators, it chiefly evaluates R&D and patent activity performance, providing separate values for university, government and business sectors, thereby offering an insight into of all three Triple Helix actors’ performance. However, the indicators are neither aggregated nor do they provide a composite measure of a country’s performance, so it might be challenging to compare, benchmark or rank countries by distinct observation of separate indicators.

Although there are multivariate approaches to Triple Helix measurement, the existing approaches neglect some important aspects. Meyer et al. (2014) highlight that “…more enriched indicators that are multi-layered and multidimensional are required to unpick the situation from different angles, thus allowing for the heterogeneity of the different actors to be voiced and heard”. Triple Helix performance measures focus more on R&D activities observed through patent and publishing activity (Leydesdorff & Meyer, 2006; Meyer et al., 2003; Xu et al., 2015). Patent activity is one of the main determinants of Triple Helix measures, since it is one of the major results of R&D activities within the innovation ecosystem (see e.g., Meyer et al., 2003; Ivanova et al., 2019). However, Baldini (2009) and Alves and Daniel (2019) stress that institutions and individuals (especially in academia) face numerous hurdles in the patenting process. Thus, this aspect should not be observed as a unique and ultimate result of Triple Helix activities. The number of spin-off companies is another R&D mechanism that may support the entrepreneurial ecosystem which is also an important indicator of the entrepreneurial level of a university (Ferri et al., 2019; Fini et al., 2017; Lawton Smith & Ho, 2006).

In the context of the Triple Helix model, support from all three actors is crucial for higher entrepreneurial intentions and the number of academic ventures (Fini et al., 2017; Samo & Huda, 2019). Nonetheless, studies and researchers have as of yet to sufficiently apply this indicator due to the limited or missing national data on the total number of spin-off companies. From another perspective, Villanueva-Felez et al. (2013) evaluated the importance of a social network and its relationship to research output (i.e., the number of research papers, books and conference papers published). Although the approach shows and evaluates the impact of interpersonal networks on research performance, it does not cover the overall performance and efficiency of a Triple Helix society. Publishing activity and a bibliometric analysis is another crucial aspect for Triple Helix collaboration (Xu et al., 2015; Priego, 2003). While Tijssen’s (2006) extensive research sheds light onto the R&D cooperation of industry and academia, it fails to incorporate a legislative perspective in order to support a holistic approach to Triple Helix measuring.

Extant approaches of measuring Triple Helix performance sometimes fail to pinpoint policy implications as a main tool for directing Triple Helix actors. Tijssen’s (2006) model, for instance, does not emphasize the importance of implications for policymakers and strategists, although the selected indicators are pertinent to the subject matter. As a further example, Egorov and Pospelova (2019) evaluated innovative activities of the Russian Arctic based on three factors: (1) the number of Russian patents granted for inventions per workforce, (2) the share of innovative goods, works and services in the total volume, and (3) the share of budget expenditures on scientific research. The results provided ranks, but they did not specify weak links and implications for all actors. Marinković et al. (2016) analyzed a broad set of multidimensional indicators, but the research only evaluated governmental performance within the Triple Helix model.

To the best of our knowledge, the most developed and applied approach to measuring Triple Helix performance thus far has been proposed by Loet Leydesdorff based on Shannon’s entropy formula as it evaluates the strength of synergy within a system based on the joint work on papers and projects. It has been further adapted and applied at a national and regional level in Germany, Norway, the Netherlands, Japan, Russia, among other countries (Leydesdorff et al., 2006; Leydesdorff & Fritsch, 2006; Leydesdorff & Sun, 2009; Leydesdorff & Strand, 2012a; b; Leydesdorff et al., 2015). The approach provides comparisons among both regions and countries and suggests a different perspective for further strategies and policies. For example, application of the mutual information Triple Helix indicator in South Korea signaled that their governmental policies failed to improve their national system by connecting actors in the field of science, technology, and industry (Park & Leydesdorff, 2010). The research provided important implications for strategists, but the conclusions were based on publication activity within the Korean innovation system. In sum, the potential of Triple Helix measurement for policy implications should be further strengthened.

Current approaches measure Triple Helix performance in the form of Triple Helix synergies and outcomes. However, there is no ready-made method to measure Triple Helix efficiency (e.g., how resources allocated to innovation can generate expected outputs). Literature review justifies focusing on the comprehensive efficiency measurement approach to improve policy. Ivanova and Leydesdorff (2015) have posed a related question: “What innovation systems are most efficient and why?” Some attempts to respond to the question have also informed our study. For instance, Mêgnigbêto (2018) used game theory to structure a model of Triple Helix relations and examine synergy indicators based on the number of papers. Tarnawska and Mavroeidis (2015) used DEA to evaluate the efficiency of 25 EU-member states based on six indicators of national innovation system performance. Another multivariate approach proposed by Jovanović et al. (2020) examines the measure of the Triple Helix synergy of 34 OECD countries through a two-step Composite I-distance method to create multivariate composite measures based on a set selected from OECD Main Science and Technology Indicators. The result was a categorization of indicators into pillars (Triple Helix actors). Jovanović et al. analyzed the performance of every pillar and the overall Triple Helix performance and rank but did not analyze the efficiency of the countries selected. Building upon the previous experience (Jovanović et al., 2020; Tarnawska & Mavroeidis, 2015), we address these issues and use DEA on a set of OECD indicators, extending the analysis using additional official data. To do so, we propose a multi-criteria efficiency approach by applying the Data Envelopment Analysis (DEA) to a dataset from 34 countries.

## The approach proposed to measure Triple Helix efficiency of innovation systems

To overcome the challenge of comprehensive performance measurement, we have designed a model to measure Triple Helix efficiency. Current approaches to measuring Triple Helix performance mainly focus on activities and outcomes, and do not fully consider the level of efficiency, in particular how input resources are efficiently used to deliver outcomes through Triple Helix interactions. Literature highlights three concepts concerning the Triple Helix interactions: spheres, spaces, and functions. Spheres refer to university, industry and government (Etzkowitz, 2008; Etzkowitz & Leydesdorff, 1995). While Etzkowitz and Leydesdorff jointly developed the Triple Helix model with a shared understanding of synergy building among the three spheres/helices, they have further elaborated on the mechanisms of Triple Helix interactions by using the concepts of spaces and functions, respectively (Leydesdorff, 2012). From a neo-institutional perspective, Etzkowitz draws attention to Triple Helix interactions of knowledge, consensus and innovation spaces, taking place in parallel with the interactions of the spheres (Etzkowitz, 2008; Etzkowitz & Zhou, 2017). From a neo-evolutionary perspective, Leydesdorff considers that the three helices also operate “as selection mechanisms asymmetrically on one another, but mutual selections may shape a trajectory as in a coevolution” (Leydesdorff, 2012, p. 28). In such a lens, the Triple Helix is perceived as three functions—namely, wealth creation, knowledge production, and normative control (Leydesdorff, 2012). In our measurement of Triple Helix efficiency, we focus on the performance of these functions.

**Fig. 1:**
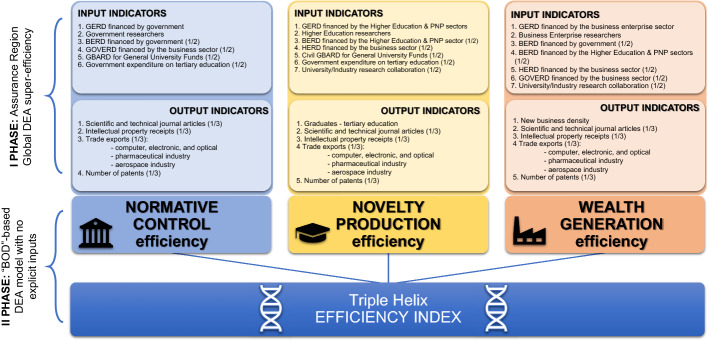
The Triple Helix efficiency index structure

As previously noted, current approaches overlook some aspects of the Triple Helix performance, so we propose a set of 19 indicators (Table 2) that offer a more comprehensive approach and address additional areas of the innovation system. The proposed model is a multi-criteria approach specifying Triple Helix functions: wealth creation, knowledge production, and normative control (Fig. 1). The model uses the efficiency approach measured by Data Envelopment Analysis (DEA). The approach examines the success of an entity (an observed unit, e.g., a country, department, sector, region, etc.) in using the provided inputs and transforming them into desired outputs (Ćujić et al., 2015). In comparison to the method developed by Leydesdorff (2003), the proposed DEA approach may imply areas to be improved for better efficiency results within the countries observed. Upon calculation, this method suggests improvements an entity should undertake to increase efficiency, improve its potential, and reach better results with the resources provided. It therefore aims to provide critical contributions and feedback to policymakers for the further development of innovation policies and technological strategies.

**Table 2:** List of selected indicators

Indicators		TH function^*^	Input/output^**^	Database^***^
1	Gross expenditure on R&D (GERD) financed by the bus. enterprise sector	WG	Input	OECD
2	GERD financed by government	WG	Input	OECD
3	GERD financed by the Higher Education and PNP sectors	NP	Input	OECD
4	Business enterprise researchers	WG	Input	OECD
5	Business expenditure on R&D (BERD) financed by government	NC-WG	Input	OECD
6	BERD financed by the Higher Education and PNP sectors	NP-WG	Input	OECD
7	Higher education expenditure (HERD) on R&D financed by the business sector	NP-WG	Input	OECD
8	Higher Education researchers	NP	Input	OECD
9	GOVERD financed by the business sector	NC-WG	Input	OECD
10	Government researchers	NC	Input	OECD
11	Civil GBARD for General University Funds	NC-NP	Input	OECD
12	Government expenditure on tertiary education	NC-NP	Input	World Bank
13	University/Industry research collaboration	NP-WG	Input	GII
14	Number of patents: "triadic" patent families; patent applications filed under the PCT	NC-NP-WG	Output	OECD
15	Graduates – tertiary education	NP	Output	World Bank
16	Scientific and technical journal articles	NC-NP-WG	Output	SCImago JR
17	Intellectual property receipts	NC-NP-WG	Output	GII
18	New business density	WG	Output	World Bank
19	Trade exports (sum of): computer, electronic and optical the pharmaceutical industry the aerospace industry	NC-NP-WG	Output	OECD

^*^Abbreviations: WG-Wealth Generation, NC-Normative Control, NP-Novelty Production

^**^Input or output indicators for a DEA model

^***^Sources: OECD—OECD (2020b), GII—Cornell University, INSEAD, and WIPO (2016), SCImago JR—SCImago JR (2020), World Bank—World Bank (2020)

In the process of designing a measure of Triple Helix efficiency, we followed the OECD framework for the creation of composite indicators (OECD, 2008): (1) developing a theoretical framework; (2) selecting variables; (3) imputation of missing data; (4) multivariate analysis; (5) normalization of data; (6) weighting and aggregation; (7) robustness and sensitivity; (8) back to the details; (9) links to other variables; and (10) presentation and dissemination.

The presented index evaluates efficiency of the selected countries based on the neo-evolutionary Triple Helix concept and its three main neo-evolutionary functions (pillars): Novelty Production (NP), Normative Control (NC) and Wealth Generation (WG) (Leydesdorff & Meyer, 2006; Leydesdorff & Zawdie, 2010). The database is comprised of four sources (Table 2): (1) OECD Main Science and Technology Indicators, (2) the Global Innovation Index (GII), 3) the SCImago Journal & Country Rank, and 4) the World Bank.

Table 2 outlines a list of the selected indicators and the source database. Indicators are classified according to the following two criteria:

1. The Triple Helix function they refer to (wealth generation, normative control and/or novelty production), and
2. Input or Output in relation to the nature of the indicator (i.e., whether it is a resource or a result) and whether it is intended to be minimized or maximized.

The indicators used in our measurement to combine multiple aspects are based on a synthesis of the literature. These indicators mainly concern R&D activities such as patents, published papers and research staff. Jovanović et al. (2020) attempted to identify and test a similar set of indicators in this paper, adding some essential aspects of innovative activity (i.e., new business density, intellectual property receipts, university and industry collaboration, and tertiary education graduates). Some input indicators are associated with two functions (informative control and wealth generation (NC-WG)), so the values of the indicators were assigned to both Triple Helix functions (1/2 of the value). As it is impossible to divide all output indicators by Triple Helix functions, the results were assigned to each Triple Helix pillar (1/3 of the value). A scheme of the Triple Helix efficiency index is given in Fig. 1.

Composite index-based performance measurement is prone to sensitive stages: availability and reliability of data, preprocessing, weighting the system and the selection of an aggregation method (Jovanović et al., 2020). To assure comprehensive data, this paper uses only reliable resources—OECD, GII, SCImago and the World Bank. An initial set consisted of 38 measures, but due to redundancy and high correlations, the final set was limited to the 19 indicators presented. Indicator values were collected for the year 2015, the last year when all data was available. Owing to reliable data and comprehensive databases, imputation was not necessary. Research results, implications and conclusions are based on the data from 2015 for 34 OECD-country members. The efficiency analysis, including normalization, weighting and aggregation were all performed through a two-phase DEA approach.

## Data envelopment analysis

Data Envelopment Analysis is an operational research non-parametric method used to evaluate the efficiency of the entities studied in decision-making units (DMUs). Charnes, Cooper, and Roads (1978) introduced this method to estimate how successful a DMU is when using multiple inputs to transform them into desired outputs (Ćujić et al., 2015). If a unit is efficient, it has an efficiency score of 1. To allow for the ranking of efficient units, it is also necessary to create a super-efficiency model (Andersen & Petersen, 1993) to calculate the exact measurements and provide efficiency scores above 1.

DEA is applicable for Triple Helix-based efficiency evaluation at a national level since this approach allows a country to achieve outstanding results, despite limited resources. This feature is especially important for smaller and less-privileged countries with restricted funds, but still able to exploit their full potential. The efficiency approach is also needed to estimate if the employed inputs result in the expected outcomes, especially in innovative activities, since practice indicates this is not usually the case (e.g., the Swedish, European and Serbian paradox) (Levi Jakšić et al., 2015).

An additional feature of the DEA method is that it allows every entity to determine the most suitable weights. As such, each DMU (for this study, *country*) can choose its own set of weights to maximize its efficiency. The feature is significant for this type of evaluation, as some countries may have superior publishing activity, but an insufficient number of patents. A unique set of weights allows units to compensate outcomes when underperforming in some of the aspects. DEA also evaluates strengths and weaknesses for every unit, provides a benchmark country and possible project improvements for more efficiency (Ćujić et al., 2015). Thus, the implications could be useful for policymakers as important input needed to create and propose national strategies.

DEA has proven superior when comparing countries from multiple, disparate perspectives:

- Technology and educational efficiency (Aristovnik, 2012; Xu & Liu, 2017)
- Innovation performance (Cai, 2011; Carayannis et al., 2015; Yesilay & Halac, 2020)
- Sustainability (Ouyang & Yang, 2020; Vierstraete, 2012; Halkos & Petrou, 2019)
- Public sector performance (Afonso et al., 2010; Baciu, & Botezat, 2014; Msann, & Saad, 2020)
- Healthcare systems (Cetin & Bahce, 2016); Top et al., 2020)
- Energy efficiency (Guo et al., 2017; Song et al., 2013; Dogan & Tugcu, 2015; Ziolo et al., 2020)

Nevertheless, the Triple Helix theory and the DEA method have not been sufficiently utilized. Tarnawska and Mavroeidis (2015) applied this method, employing six indicators at most, which is an insufficient number for such a complex problem as knowledge triangle policy in the EU countries. Our research aims to introduce a comprehensive measure of Triple Helix-based efficiency, for which we provide a detailed model structure in the following section. The research involves a set of 34 OECD countries and a selected set of 19 indicators. The results will compare the efficiency of OECD countries based on the cooperation between the three pillars.

## The two-phase DEA approach

DEA has proven to be a useful method when constructing a composite index due to its specific characteristics (Cherchye et al., 2008) in which individual indicators are aggregated free of a predefined set of weights. This allows each unit observed to determine its own weighting system. Every assessed entity also takes into consideration the performance of the other entities observed, which is known as the “benefit of the doubt—BOD” approach (Cherchye et al., 2007; Savić & Martić, 2017). DEA-based composite indices are proven to be an effective tool for the evaluation and comparison of entities from disparate perspectives: logistic performance, sustainability, human development and eco-efficiency as well as company performance (see e.g. Mariano et al., 2017; Halkos et al., 2016; Shi & Land, 2020; Huang et al., 2018; Dutta et al., 2020).

A number of DEA mathematical model formulations may be applied depending on the type of the problem examined (e.g., input-oriented, output-oriented, BCC, CCR, undesired outputs, BOD, hierarchical approach) (Paradi et al., 2018). This paper uses the two-phase approach to construct a composite measure of Triple Helix-based efficiency.

In the first phasea, indicators were aggregated within each Triple Helix pillar and the scores were provided by a combination of Assurance Region Global (Cooper et al., 2007) and DEA super-efficiency models:

1 $$\begin{aligned} &(\mathrm{max}){h}_{k}={\sum }_{r=1}^{s}{u}_{r}{y}_{rk} \hfill \\ &{\sum }_{i=1}^{m}{v}_{i}{x}_{ik}=1 \hfill \\ &{\sum }_{r=1}^{s}{u}_{r}{y}_{rj}-{\sum }_{i=1}^{m}{v}_{i}{x}_{ij}\le 0, j=1, 2, ....., n,j\ne k\end{aligned}$$

$$lb\le \frac{{v}_{i}{x}_{ik}}{{\sum }_{i=1}^{m}{v}_{i}{x}_{ij}}\le ub, i=1, 2, ....., m$$

$${u}_{1},...,{u}_{s}\ge 0,{v}_{1},...,{v}_{m}\ge 0$$

where *n* is the number of DMUs – countries (j = 1,…n); *m* – the number of inputs (i = 1,…,m); *s* – the number of outputs (r = 1,…,*s*); ${x}_{ij}$ – the known amount of *i* − the input of DMU*j* (${x}_{ij}$> 0, *i* = 1,2,…,*m*, *j* = 1,2,…,*n*); ${y}_{rj}$ – the known amount of *r* − the output of the DMU*j* (${y}_{rj}$> 0, *r* = 1,2,…,*s*, *j* = 1,2,…,*n*); ${h}_{k}$ (*k* = 1,…,*n*) – the efficiency score; ${v}_{i}$ (*i* = 1,…,*m*) – the weight assigned to *i* − the input by the DMU*k*; ${u}_{r}$ (r = 1,…,s) – the weight assigned to *r* − the input by the DMU*k*; and the range [*lb*,*ub*] signifies the influence of all inputs into the total weighted input. This model provides relatively efficient scores and ranks by comparing countries within the studied set of 34 OECD countries, for each pillar of wealth generation, normative control and novelty production.

In the second phase, the pillar scores were aggregated for every country through a “BOD”-based DEA model that had no explicit inputs (Cherchye et al., 2007):

2 $$\begin{aligned} & (\mathrm{max})ef{f}_{k}={\sum }_{r=1}^{s}{\overline{u}}_{r}{h}_{rk} \hfill \\ & \sum_{r=1}^{s}{\overline{u} }_{r}{h}_{rj}\le 0, j=1, 2, \dots ., n, j\ne k \hfill \\ & {\overline{u}}_{1},...,{\overline{u}}_{s}\ge 0 \end{aligned}$$

where *n*–the number of DMUs (countries), *s*–the number of pillars; ${h}_{rj}$–the efficiency score obtained in the previous phase for *r* − the pillar (${y}_{rj}$> 0, *r* = {1,2,3}, *j* = 1,2,…,*n*); ${eff}_{k}$ (*k* = 1,…,*n*) –the efficiency score of the DMU*k*; ${\overline{u} }_{r}$(*r* = 1,…,*s*) –the weight assigned to r − the output by the DMU*k*.

Based on the two models presented, the efficiency of each sub-index was calculated, thus obtaining the efficiency scores for each Triple Helix function. The values of the sub-indices and the second DEA model, as well as the overall Triple Helix super-efficiency of the countries selected, provided a country ranking based on the scores yielded.

## Measuring the Triple Helix-based efficiency of OECD countries

**Fig. 2:**
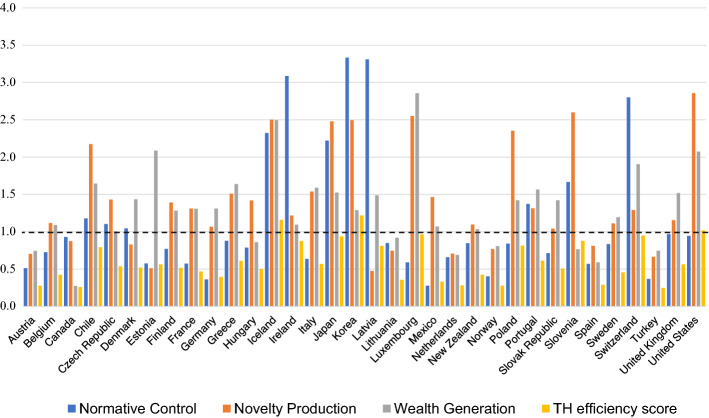
Triple Helix super-efficiency scores

Having evaluated the efficiency of each Triple Helix pillar, Table 4 presents the efficiency of the selected countries using a specified efficiency measure within each Triple Helix function, as well as the overall Triple Helix efficiency. Appendix A-C provides a detailed calculation and impact of the indicators for every country. Figure 2 gives a graphical overview of the scores in Table 4, which enables visual comparison of country scores. The values in the Appendix represent data-driven weights illustrating the importance of each input and output indicator informing the efficiency score. The value of the efficiency score specifies if a country has been efficient. When the entity has an efficiency score equal to or higher than 1, it is considered efficient, wherein the given resources result in a high outcome level. Table 3 provides the results of descriptive statistics for all three pillars: the number of efficient countries, the maximum, minimum and average efficiency as well as the standard deviation of the efficiency scores.

**Table 3:** Descriptive statistics of DEA efficiency

Variables	Normative Control	Wealth Generation	Novelty Production	Triple Helix
Number of efficient countries	10	25	24	3
Maximum super-efficiency	3.333	2.857	0.474	1.310
Minimum super-efficiency	0.278	0.274	2.857	0.150
Average super-efficiency	1.229	1.317	1.400	0.553
Standard deviation of super-eff. scores	0.886	0.542	0.686	0.273

**Table 4:** Efficiency scores and ranks

Countries	Normative control			Novelty production			Wealth generation			Eff. score	Rank
	Eff. score	Rank	Weight	Eff. score	Rank	Weight	Eff. score	Rank	Weight		
South Korea	3.333	1	0.8824	2.496	5	0.2877	1.291	18	0.0500	**1.220**	**1**
Iceland	2.323	5	0.3478	2.502	4	0.1661	2.495	2	0.6481	1.162	2
USA	0.945	1	0.0500	2.857	1	0.8796	2.075	4	0.0889	1.018	3
Luxembourg	0.590	26	0.0500	2.553	3	0.0500	2.857	1	0.8636	0.964	4
Switzerland	2.802	4	0.5732	1.291	17	0.0500	1.905	5	0.3266	0.950	5
Japan	2.222	6	0.1348	2.479	6	0.7288	1.525	10	0.0754	0.939	6
Slovenia	1.667	7	0.0893	2.599	2	0.7406	0.767	29	0.0500	0.890	7
Ireland	3.087	3	0.7767	1.217	18	0.0500	1.095	21	0.0500	0.877	8
Poland	0.843	18	0.0500	2.354	7	0.7041	1.421	15	0.0611	0.815	9
Latvia	3.310	2	0.5528	0.474	34	0.0500	1.489	12	0.2078	0.811	10
Chile	1.180	9	0.0716	2.175	8	0.6396	1.645	6	0.0814	0.793	11
Portugal	1.374	8	0.0885	1.315	15	0.0500	1.565	9	0.4737	0.612	12
Greece	0.880	15	0.0573	1.511	10	0.0500	1.639	7	0.5032	0.610	13
Italy	0.638	25	0.0500	1.539	9	0.0500	1.590	8	0.4696	0.570	14
UK	0.970	12	0.0617	1.157	19	0.0500	1.518	11	0.4528	0.565	15
Estonia	0.576	27	0.0500	0.512	33	0.0500	2.088	3	0.4637	0.564	16
Czech Rep	1.105	10	0.0669	1.432	12	0.4209	1.007	25	0.0500	0.538	17
Denmark	1.046	11	0.0638	0.830	26	0.0500	1.435	13	0.4069	0.521	18
Finland	0.772	21	0.0500	1.392	14	0.3991	1.283	19	0.0679	0.517	19
Slovak Rep	0.715	23	0.0500	1.041	24	0.0500	1.422	14	0.4089	0.509	20
Hungary	0.789	20	0.0500	1.420	13	0.4020	0.863	27	0.0500	0.502	21
France	0.575	28	0.0500	1.311	16	0.0500	1.308	17	0.3682	0.468	22
Sweden	0.834	19	0.0528	1.113	21	0.0500	1.196	20	0.3548	0.458	23
New Zealand	0.847	17	0.0500	1.097	22	0.3202	1.034	24	0.0550	0.425	24
Belgium	0.727	22	0.0500	1.118	20	0.3125	1.091	22	0.0615	0.424	25
Germany	0.362	33	0.0500	1.069	23	0.0500	1.312	16	0.2956	0.396	26
Lithuania	0.850	16	0.0510	0.747	29	0.0500	0.921	26	0.2559	0.357	27
Mexico	0.278	34	0.0500	1.465	11	0.1207	1.070	23	0.1612	0.332	28
Spain	0.571	29	0.0500	0.813	27	0.1904	0.592	33	0.0500	0.290	29
Netherlands	0.659	24	0.0579	0.708	30	0.1740	0.690	32	0.0500	0.282	30
Norway	0.404	31	0.0500	0.770	28	0.0500	0.808	28	0.1782	0.278	31
Austria	0.512	30	0.0500	0.703	31	0.0500	0.744	31	0.1776	0.278	32
Canada	0.929	14	0.1607	0.875	25	0.0500	0.274	34	0.0500	0.261	33
Turkey	0.368	32	0.0500	0.666	32	0.0500	0.746	30	0.1486	0.249	34

A total of 10 countries are efficient in terms of normative control performance (efficiency score > 1). The results of the first phase indicate the most efficient countries to be South Korea, Iceland and Latvia. South Korea’s high score is based on its respectively high level of patent activity (0.9554). Latvia also shows strong publishing activity (0.7803) in addition to somewhat significant trade exports (0.1123) and patents (0.1024). Iceland is marked as efficient due to its exceptional intellectual property receipts (0.9850) in comparison to invested resources. Conversely, Mexico, Germany, and Turkey all have low efficiency scores (0.278, 0.362, and 0.368, respectively). Turkey has high government investments in GERD but does not sufficiently commercialize intellectual property (0-weight) as well as trade exports (0.005). Similarly, Mexico scores well on published papers (0.6450) and exports (0.3490), but is brought down by its low number of patents and intellectual property receipts. It may come as a surprise due to its high investments, but Germany’s insufficient outcomes result in it being ranked as inefficient. Although Germany is strongest in patent activity (0.6782) and trade exports (0.2947) for innovative activity, these indicators insufficiently compensate for its low commercialization of intellectual property and its low number of published papers.

The results show there to be 25 efficient countries in the wealth generation pillar among the innovative systems examined. Most of the countries studied show an efficient function, where the average efficiency score is 1.317 with a standard deviation of 0.542. The most efficient are Luxembourg (2.857), Iceland (2.495), and Estonia (2.088). Luxembourg scores high due to its 2 innovative outputs: new business density (0.4660) and intellectual property receipts (0.4881), while Estonia has an astonishing new business density as a leading innovative output, followed by a modest number of scientific articles. Iceland again owes its high rank to intellectual property receipts (0.8682), but its new business density also plays an important role in their innovative output (0.1168). On the other hand, Canada (0.274), Spain (0.592) and the Netherlands (0.690) all score the lowest, which may come as a surprise. Whereas Canada does present high patent activity, it is insufficient to compensate for its other outputs and their activity does not follow the investments provided. Spain’s innovative activity output is based solely on scientific articles, while its intellectual property receipts are so low that they only gain a 0-weight, unable to contribute to a better efficiency score. Similarly, the Netherlands scores no weight for intellectual property, signifying that this factor should be improved through policy intervention.

Evaluating novelty production performance shows there to be 24 efficient countries. As in the wealth generation pillar, most are efficient, achieving a high average efficiency score of 1.400 with a standard deviation 0.686. The United States (2.857), Slovenia (2.599) and Luxembourg (2.553) have the most efficient universities in relation to innovative activity. Although the United States presents outstanding university output for all indicators, its level of intellectual property receipts has the highest impact (0.98) in comparison to the other countries examined. Slovenia scores a balanced innovative output mainly focused on patents (0.2613), journal articles (0.2344) and intellectual property receipts (0.4943). In contrast, the results show that Latvia (0.474), Estonia (0.512), and Turkey (0.666) have the least efficient knowledge creation sector. Even though Latvia and Estonia do have a substantial number of scientific articles, their number of graduates and intellectual property receipts is not sufficiently high in comparison to their investments. With a low value of university/industry collaboration, Turkey scored the lowest with a 0-weight for intellectual property receipts.

In terms of the Triple Helix super-efficiency index, the results of the second phase analysis show only three countries to be efficient: South Korea (1.220), Iceland (1.162) and the USA (1.018). The remaining countries fall below the efficiency frontier. South Korea, wealth generation not being a crucial factor (0.050), achieves its high score mainly due to its strong normative control results (0.882) and moderate novelty production (0.288). The United States bases its high score on its strong knowledge creation performance (0.8795), while Iceland, leading with wealth creation (0.6481) and normative control (0.3478), has a more balanced Triple Helix functions’ efficiency. Turkey (0.2486), Canada (0.2607) and Austria (0.2776) score the least efficient Triple Helix-based innovation systems. In comparison to the other countries studied, Turkey has highly inefficient normative control and novelty production (0.050), possessing only slightly stronger wealth-generation performance (0.1486). Canada is similar in its weak novelty creation and normative control (0.0500), although it does present a stronger legislative function (0.1607).

## Discussion and implications

The results present provide six important implications for the proposed model.

**Fig. 3:**
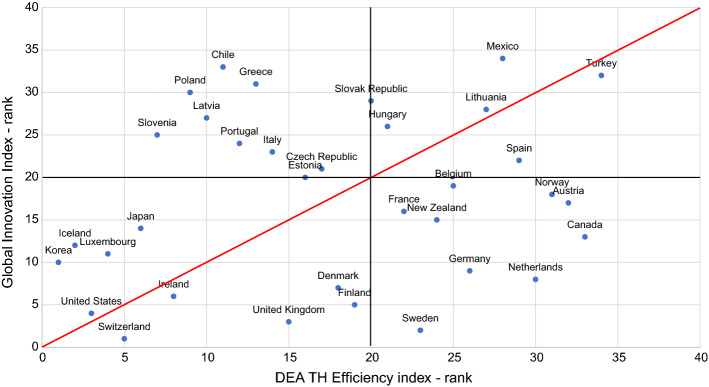
The Triple Helix innovation matrix

1. The most competitive advantage of DEA is its variable set of weights, allowing for every unit to compensate for indicators which may be used to rank the results. As for the efficiency of legislative function, the top three countries have their high scores rooted in separate aspects: Ireland’s high trade exports, Latvia’s outstanding level of published articles and trade exports, and South Korea’s superior number of patents. Estonia emerged as the 3rd ranked country by wealth generation efficiency due to its high intensity of newly established businesses, which is another example of DEA’s advantage of taking into consideration the competitiveness of an innovative ecosystem. Japan, for example, focuses its efforts on patents, Iceland is excellent in charging for the use of its intellectual property, while Estonia creates new business ventures. South Korea’s superiority in innovative performance has already been confirmed by Bloomberg’s Innovation Index methodology – ranked 1st (Bloomberg, 2020, 2016), and Global Innovation Index (Cornell University, INSEAD, and WIPO, 2016) – ranked 11^th^.
2. The approach presented in this paper evaluates if a country is efficient – exploiting its invested resources possibly resulting in an appropriate output. Despite its remarkable innovative output, Germany is surprisingly not highly ranked. These results imply that Germany might achieve higher scores considering its resources and investments. Likewise, France’s high GOVERD financed by the business sector has not resulted in high outputs within the normative control function.
3. Descriptive statistics in Table 3 indicates the greatest deviations in the normative control sector, which has also been shown as the least efficient. As expected, the main role of this sector is to provide sufficient funds and legislative support; the main role of creator of innovative outputs, on the other hand, is dedicated to wealth generation and novelty production functions.
4. In comparison to the Global Innovation Index aggregation method, the presented approach considers the size of a country and its available resources. Figure 3 highlights these differences, comparing the normalized GII (in comparison to the countries studied) and the Triple Helix super-efficiency rank. Beneath the red line are those countries that have a higher GII score than their Triple Helix super-efficiency score, while above the red line are those countries that have a higher Triple Helix super-efficiency score in comparison to the GII.
  The first (bottom left) quadrant represents those countries successful by both criteria, while the second (bottom right) lists countries that are successful innovators, but not efficient from a Triple Helix functions perspective. The top left quadrant shows Triple Helix efficient countries that maximize the utilization of their resources but are not listed in the top national innovative systems according to GII methodology. The top right quadrant shows the countries with a lower rank in both scores. The closer the countries are to the top right corner of the quadrant, the less innovative they are according to both criteria. This matrix might prove to be a valuable visual tool for policymakers.
  According to Fig. 3, the United States is the best among all the countries examined, while Switzerland also achieved significant results. Were Switzerland to improve their novelty production efficiency (scoring the lowest weight in the Triple Helix index – 0.050) they could upgrade their position in this innovation matrix. Moreover, some countries, such as Estonia, are successful in the utilization of their resources but do not follow the same trend in GII, which might imply higher potential should additional resources be invested. This result is especially important for highly efficient countries, such as Iceland, Luxembourg, Japan, and South Korea, all of which have a high potential to grow even more innovative with added resources. On the other hand, countries such as Sweden, the Netherlands, the United Kingdom, Germany and Canada must create higher outputs to justify their resources, such as with Estonia. For example, Germany’s novelty production and legislative function efficiency is surprisingly low, as well as the Netherlands’ wealth generation and Canada’s normative control.
5. Aparticular quality of the DEA method is that it provides feedback on improvements that should be made within an entity in order for it to achieve a higher level of efficiency (Paradi et al., 2018). The results help identify weak links within each innovation system, providing the exact measure of the improvement that an entity should make to become efficient. For policymakers, this advantage could be a crucial contribution, applicable when determining the need to improve measures and policies within the system examined.
  The results of this method may be used as a direct input for a national-level decision-making process to improve the performance of Triple Helix pillars. The proposed model is scalable and, with proper data collection, could be applicable regionally or locally. Such an approach should indicate where countries stand from a Triple Helix functions perspective and provide further steps to be taken for a higher level of innovativeness within their system. Low weighting scores for a number of the outputs in Appendix A-C indicate where improvements could be made. Policymakers could use their expertise to target these quantitative results to forge better policies that may strengthen their national system’s innovativeness and efficiency.
6. The results provide insight into the European Innovation Paradox. The DEA Triple Helix score presented in Fig. 3 clearly points to lower innovation systems’ efficiency for European countries within the innovation efficiency rankings. The new proposed Triple Helix-based efficiency measurement tool has indicated the domains of inefficiency specifically related to the Triple Helix functions: normative control, novelty production, wealth generation. It clearly lays out the need to develop and implement policy measures towards the interconnectivity and harmonization of the said factors. Listed below are five factors that help account for the innovation paradox as indicated by the results of the Triple Helix efficiency measurements:
7. The “Valley of Death” phenomena within the innovation cycle (Beard et al., 2009), with losses of innovation potential in phases of innovation transition from ‘discoveries to ideas to implementation and diffusion’ due to the limited efficiency of the Triple Helix actors involved in innovation creation (Research), innovation implementation and diffusion (Development).
8. A comprehensive approach not sufficiently established and implemented that may incorporate the corresponding complements to investments necessary to achieve complex sustainable development returns.
9. Weak managerial and organization practices, as key firm capabilities, to bring innovation successfully to the market especially for new emerging technology and innovation entrepreneurial ventures.
10. Ineffective innovation policies not finely tuned to the characteristics of concrete innovation ecosystems.
11. Weak government capabilities in developing and implementing effective innovation policies.

The analysis presented provides thorough and substantial answers to the research questions posited. The results firmly establish the opportunity of developing an efficiency-based Triple Helix composite index. This approach allows smaller countries to excel even under limited resources when used efficiently. In addition, the research illustrates potential implications for policy makers where additional expertise may improve certain national environments. Nevertheless, there are prerequisites for the DEA method: sufficient observation units, available indicators, non-negative data and classification according to the Triple Helix agents. This paper proposes a framework for measuring Triple Helix-based efficiency. With an updated set of indicators, the solution is scalable at any level (i.e., national, regional, local) and is useable to measure unit efficiency within each Triple Helix pillar (government, university or industry) with the data available.

## Conclusion

The presented research and results point to a novel approach to creating a composite index for Triple Helix-based efficiency evaluation. Leydesdorff and Ivanova (2016) have highlighted that the Triple Helix model has become neo-evolutionary in relation to interactions among selection environments as determined by demand, supply and technological capabilities. The approach presented in this paper supports this claim and considers the technological capabilities (inputs) of the selected environment (a country) and evaluates the results obtained (outputs) in comparison to the available resources of a national innovation system.

In response to the first research question, we have summarized and evaluated approaches to measuring Triple Helix performance (Table 1). The analysis identified gaps in existing methodologies, which in turn served as a foundation to propose a novel application of DEA. Answering the second research question is an original methodology aimed at introducing a holistic, systemic approach to measuring Triple Helix-based efficiency of innovation systems. A combined set of indicators from verified official databases is classified into three separate pillars building up a comprehensive composite index of a Triple Helix-based innovation ecosystem.

To estimate the innovation efficiency of the 34 OECD countries examined, a multilevel DEA model was applied. The findings imply the possibility of creating a comprehensive measure of Triple Helix efficiency at the national level that may provide performance scores for all Triple Helix functions: Novelty Production, Normative Control and Wealth Generation, as well as an overall Triple Helix index based on the scores of these three pillars. The outcomes presented provide valuable data on weak links within an ecosystem and the improvements that could be made to create a more innovative and efficient national system based on the examination of indicators within the pillars. The measurement findings point to multiple important factors to be considered more as accounted for the European Innovation Paradox: weak governmental capacities for policy implementation, ineffective and unadjusted innovation policies, weak managerial practices, lack of comprehensive approaches and practices to utilize investment, and loss of innovation potential that may be attributed to the limited efficiency of the Triple Helix actors involved in both research and development activities.

This study provides three main scholarly contributions: (1) a summary and critical analysis of approaches to measuring the Triple Helix; (2) a further utilizable application of the Data Envelopment Analysis method as well as a demonstration of its functionality; and (3) a step towards measuring the Triple Helix-based performance nationally.

While this study provides an innovative and useful approach to measuring Triple Helix-based efficiency, there are five distinct limitations that must be acknowledged:

1. The method is sensitive to the number of indicators in the model. In DEA, the number of indicators is determined by the total number of units (for the purpose of our research, countries). In general, the multiplied score of both the inputs and outputs should be the minimum of the countries examined (e.g., four input and five output indicators require at least 20 countries). As this paper uses a two-phase approach and aggregates indicators into pillars, this limitation is mitigated.
2. The set of indicators presented may be expanded, as data important as markers of innovation activity were unavailable. The data in this research was manly collected from the OECD’s Main Science and Technology indicators, providing official, functional and available data. However, numerous important aspects may not be covered by these indicators. For instance, a crucial factor of a university’s innovative performance is its number of spin-off companies. Unfortunately, to the best of our knowledge, there are no publicly available national data. This indicator would enhance a holistic approach and improve the reliability of the results provided. This limitation could be resolved by adapting methodologies and instructions for measuring innovative performance at a national level.
3. The weights presented are data-driven, based on the presented indicator values. If data or a country is excluded from the ranking, the values and efficiency scores might change.
4. The boundaries provided for the weight values are subject to debate and might be changed in a what-if analysis to provide more reliable information. In this research, no zero-weights were permitted for any indicator, but the limitations were not strict, following the theoretical approach of the Triple Helix concept, in which actors may take one another’s roles within the ecosystem (Cai & Etzkowitz, 2020).
5. For those indicators that affect two or three helices, indicator values were arbitrarily assigned (equally distributed).

To further improve the measurement and its applications, particularly to overcome the hitherto noted limitations, further studies are to be carried out. Future research will assess an updated set of indicators, by which we will attempt to identify novel metrics necessary to shed light onto all important perspectives of Triple Helix-based innovative activity. In order to compare the results with the most renowned approach in this field, mutual information Triple Helix indicator, it would be useful to calculate the mutual information indicator for examined OECD countries. This indicator provides valuable conclusions for policymakers, while the presented efficiency approach could offer additional information and a different perspective of implications that would be significant for strategies. We shall also test alternative DEA models and a modified set of weights to identify the most relevant approach to measuring Triple Helix-based efficiency. Network DEA is suitable for the Triple Helix perspective, as it may include indicators of inputs and outputs created individually or mutually by actors. That way, the interactions between the Triple-Helix actors may also be covered.

Finally, the applicability of the model will be tested at other levels (regional and local) to assess the scalability of the model and derive additional applications of the proposed framework. Furthermore, modification similar to Fair DEA (Radovanović et al., 2021) model may be implied to eliminate disparate impact on efficiency measure between the developed and undeveloped units.

With proper model adjustments and the domain expertise of local strategists and analysts, the model presented might prove to be a valuable tool for policymakers by providing essential results through an approach scalable at national, regional and local levels.

## Data Availability

The datasets analysed during the current study are available in the OECD repository, https://stats.oecd.org/Index.aspx?DataSetCode=MSTI_PUB, World Bank repository, https://databank.worldbank.org/source/education-statistics-%5e-all-indicators, SCImago JR repository, https://www.scimagojr.com/countryrank.php, and Global Innovation Index repository, https://www.wipo.int/edocs/pubdocs/en/wipo_pub_gii_2016.pdf. The datasets generated during the current study are available from the corresponding author on reasonable request.

## Appendix A *Normative Control* sub-index performance

**Table:**

	Inputs						Outputs				Results	
	GERD financed by govt	Gov. researchers	BERD financed by govt	GOVERD financed by bus	Civil GBARD for gen. uni. funds	Gov. expenditure on tert. edu	Number of patents	Scientific & technical articles	Intellectual property receipts	Trade exports	Efficiency	Gov. rank
Austria	0.3558	0.1791	0.0500	0.0500	0.1110	0.6000	0.8051	0.1849	0.0050	0.0050	0.743	23
Belgium	0.3793	0.0500	0.0500	0.0500	0.0568	0.6000	0.5803	0.0953	0.3195	0.0050	0.843	21
Canada	0.0500	0.0500	0.3732	0.3378	0.1765	0.0500	0.2112	0.0050	0.7788	0.0050	0.964	14
Chile	0.0500	0.0500	0.1468	0.0500	0.0733	0.0500	0.9850	0.0050	0.0050	0.0050	2.381	5
Czech Rep	0.0500	0.0500	0.0500	0.1241	0.1612	0.6000	0.0050	0.0050	0.9850	0.0050	0.966	13
Denmark	0.0500	0.0500	0.1610	0.4419	0.0500	0.0500	0.9420	0.0480	0.0050	0.0050	1.246	10
Estonia	0.6000	0.6000	0.0500	0.0500	0.0000	0.3653	0.1904	0.0469	0.7577	0.0050	0.600	29
Finland	0.1947	0.0535	0.0869	0.0500	0.1358	0.6000	0.5640	0.1170	0.2804	0.0386	0.892	19
France	0.3320	0.1945	0.0500	0.0500	0.3707	0.6000	0.6720	0.2606	0.0625	0.0050	0.626	28
Germany	0.5559	0.6000	0.6000	0.0500	0.6000	0.6000	0.2813	0.7087	0.0050	0.0050	0.333	33
Greece	0.0500	0.0500	0.3364	0.0500	0.0500	0.6000	0.0050	0.0050	0.9822	0.0078	0.880	20
Hungary	0.2663	0.0500	0.0500	0.2003	0.1630	0.6000	0.6091	0.0583	0.2425	0.0902	0.752	22
Iceland	0.0500	0.0500	0.0500	0.0500	0.0500	0.0500	0.0050	0.0278	0.0211	0.9461	3.333	2
Ireland	0.0500	0.0500	0.0500	0.1409	0.1211	0.0500	0.0882	0.0050	0.5457	0.3611	2.164	7
Italy	0.0500	0.0500	0.6000	0.1420	0.1495	0.6000	0.0050	0.1161	0.8739	0.0050	0.628	26
Japan	0.0500	0.0500	0.0500	0.0500	0.0894	0.1467	0.0050	0.9850	0.0050	0.0050	2.293	6
Korea	0.0500	0.0500	0.0500	0.0500	0.0500	0.0500	0.0050	0.9571	0.0050	0.0329	3.333	1
Latvia	0.0500	0.0500	0.0525	0.0500	0.0500	0.0500	0.0050	0.0050	0.9839	0.0061	3.306	3
Lithuania	0.0500	0.0500	0.0915	0.2665	0.0500	0.6000	0.6357	0.0050	0.3487	0.0107	0.903	18
Luxembourg	0.0500	0.0500	0.3120	0.2472	0.1929	0.6000	0.5942	0.0842	0.1447	0.1769	0.689	24
Mexico	0.6000	0.6000	0.6000	0.6000	0.6000	0.6000	0.9072	0.0867	0.0050	0.0011	0.278	34
Netherlands	0.6000	0.6000	0.0500	0.0500	0.1952	0.0500	0.0050	0.3206	0.6744	0.0000	0.647	25
New Zealand	0.4305	0.1533	0.0500	0.0500	0.0500	0.3163	0.2874	0.0794	0.6191	0.0141	0.952	15
Norway	0.2040	0.2005	0.6000	0.0500	0.1380	0.6000	0.6056	0.0638	0.3256	0.0050	0.558	31
Poland	0.3141	0.0500	0.0500	0.0760	0.0674	0.4978	0.7940	0.0050	0.1960	0.0050	0.948	16
Portugal	0.0500	0.0500	0.2535	0.0818	0.1589	0.0962	0.0050	0.0050	0.9850	0.0050	1.449	9
Slovak Rep	0.0913	0.0500	0.6000	0.2059	0.0500	0.6000	0.9850	0.0050	0.0050	0.0050	0.626	27
Slovenia	0.3331	0.0500	0.0500	0.0500	0.0684	0.0500	0.0050	0.0775	0.7117	0.2058	1.663	8
Spain	0.0500	0.6000	0.0519	0.0500	0.3964	0.6000	0.0050	0.0935	0.9015	0.0000	0.572	30
Sweden	0.2834	0.0500	0.0500	0.0500	0.0500	0.6000	0.6029	0.1619	0.2302	0.0050	0.923	17
Switzerland	0.0500	0.1073	0.0500	0.0500	0.0500	0.0500	0.0050	0.6819	0.2971	0.0160	2.799	4
Turkey	0.0881	0.6000	0.0500	0.0500	0.6000	0.6000	0.9001	0.0949	0.0050	0.0000	0.503	32
UK	0.6000	0.0838	0.0500	0.0500	0.0500	0.1761	0.0050	0.1857	0.8043	0.0050	0.990	12
USA	0.0500	0.0500	0.0500	0.1999	0.5815	0.0750	0.0050	0.9850	0.0050	0.0050	0.994	11

## Appendix B *Wealth Creation* sub-index performance

**Table:**

	Inputs							Outputs					Results	
	GERD financed by bus	Bus. researchers	BERD financed by gov	BERD financed by HE	HERD financed by bus	GOVERD financed by bus	Uni/ind. collaboration	New bus. density	No. of patents	Scient. &technical articles	Intellect. property receipts	Trade exports	Effici-ency	Ind. rank
Austria	0.0500	0.2576	0.0500	0.4539	0.0500	0.0555	0.4265	0.0050	0.4990	0.4250	0.0050	0.0660	0.744	31
Belgium	0.0500	0.0500	0.0500	0.2218	0.0500	0.0500	0.4447	0.0050	0.4268	0.3999	0.0050	0.1633	1.091	22
Canada	0.6000	0.6000	0.6000	0.0500	0.6000	0.6000	0.6000	0.0050	0.9800	0.0050	0.0050	0.0050	0.274	34
Chile	0.0500	0.3078	0.0500	0.0500	0.0500	0.0500	0.0500	0.0289	0.2160	0.7452	0.0050	0.0050	1.645	6
Czech Rep	0.0500	0.0500	0.0500	0.2367	0.0500	0.0500	0.5068	0.0461	0.0050	0.7646	0.0050	0.1793	1.007	25
Denmark	0.0500	0.0500	0.0532	0.0500	0.1698	0.1275	0.1963	0.0050	0.4827	0.5023	0.0050	0.0050	1.435	13
Estonia	0.0500	0.0500	0.0500	0.0500	0.0500	0.1790	0.0500	0.7900	0.0050	0.1950	0.0050	0.0050	2.088	3
Finland	0.0500	0.0500	0.3415	0.1880	0.0500	0.0500	0.0500	0.0050	0.9800	0.0050	0.0050	0.0050	1.283	19
France	0.0500	0.0500	0.0500	0.1822	0.0876	0.0500	0.2950	0.0050	0.0050	0.5034	0.0908	0.3958	1.308	17
Germany	0.0500	0.0500	0.1823	0.0500	0.0500	0.0500	0.3301	0.0050	0.0426	0.2302	0.0050	0.7171	1.312	16
Greece	0.0500	0.0697	0.0500	0.0644	0.0500	0.0500	0.2761	0.0050	0.0050	0.9800	0.0050	0.0050	1.639	7
Hungary	0.3094	0.0500	0.0500	0.0500	0.0500	0.0500	0.6000	0.0050	0.0050	0.3605	0.6245	0.0050	0.863	27
Iceland	0.1008	0.0500	0.0500	0.0500	0.0500	0.0500	0.0500	0.1168	0.0050	0.0050	0.8682	0.0050	2.495	2
Ireland	0.0500	0.0500	0.0500	0.0000	0.0500	0.1129	0.6000	0.0050	0.3336	0.4699	0.0050	0.1865	1.095	21
Italy	0.0500	0.0500	0.0500	0.0500	0.1368	0.0500	0.2420	0.0050	0.0444	0.8807	0.0649	0.0050	1.590	8
Japan	0.0500	0.0500	0.0500	0.0500	0.0500	0.0500	0.3557	0.0050	0.9800	0.0050	0.0050	0.0050	1.525	10
Korea	0.0500	0.0500	0.0500	0.2463	0.0500	0.1455	0.1829	0.0074	0.2195	0.3921	0.0050	0.3761	1.291	18
Latvia	0.0500	0.0500	0.0500	0.0000	0.1142	0.3453	0.0622	0.2623	0.1112	0.5105	0.0212	0.0948	1.489	12
Lithuania	0.0500	0.0500	0.2397	0.0500	0.0500	0.0500	0.5955	0.0050	0.0050	0.9170	0.0680	0.0050	0.921	26
Luxembourg	0.0500	0.0500	0.0500	0.0500	0.0500	0.0500	0.0500	0.4660	0.0359	0.0050	0.4881	0.0050	2.857	1
Mexico	0.0500	0.0500	0.0500	0.0500	0.2514	0.0500	0.4330	0.0050	0.0050	0.5901	0.0050	0.3949	1.070	23
Netherlands	0.0500	0.0500	0.0500	0.0500	0.0500	0.6000	0.6000	0.0673	0.1224	0.4528	0.0000	0.3575	0.690	32
New Zealand	0.4497	0.0500	0.0500	0.0500	0.1124	0.0500	0.2050	0.2736	0.4452	0.2566	0.0197	0.0050	1.034	24
Norway	0.2652	0.0500	0.0500	0.1721	0.0500	0.0500	0.6000	0.2029	0.3519	0.4269	0.0050	0.0133	0.808	28
Poland	0.1099	0.0500	0.0500	0.1287	0.0500	0.0500	0.2649	0.0050	0.0050	0.9760	0.0050	0.0090	1.421	15
Portugal	0.0500	0.0500	0.1824	0.0500	0.0500	0.0500	0.2067	0.0050	0.0050	0.9800	0.0050	0.0050	1.565	9
Slovak Rep	0.0500	0.1987	0.0500	0.0730	0.2313	0.0500	0.0500	0.0233	0.0050	0.9617	0.0050	0.0050	1.422	14
Slovenia	0.0500	0.0500	0.4312	0.2119	0.2985	0.2121	0.0500	0.0222	0.0050	0.9628	0.0050	0.0050	0.767	29
Spain	0.0500	0.1646	0.0500	0.5561	0.0500	0.2192	0.6000	0.0815	0.0900	0.6325	0.0000	0.1960	0.592	33
Sweden	0.0500	0.0500	0.0500	0.0500	0.1466	0.0500	0.4398	0.0361	0.0862	0.2610	0.6117	0.0050	1.196	20
Switzerland	0.0500	0.1810	0.0500	0.0500	0.0500	0.0939	0.0500	0.0050	0.8103	0.0050	0.0050	0.1747	1.905	5
Turkey	0.0500	0.0500	0.1810	0.1449	0.0500	0.2640	0.6000	0.0405	0.0499	0.8671	0.0000	0.0425	0.746	30
UK	0.1169	0.0500	0.0500	0.0500	0.0500	0.1122	0.2296	0.1356	0.0050	0.6599	0.0050	0.1945	1.518	11
USA	0.0500	0.0500	0.0500	0.0500	0.0500	0.0500	0.1819	0.0050	0.1023	0.8827	0.0050	0.0050	2.075	4

## Appendix C *Novelty Production* sub-index performance

**Table:**

	Inputs							Outputs					Results	
	GERD finan. by Higher Edu	Higher Edu. researchers	BERD financed by the High. Edu	HERD financed by bus. sector	Civil GBARD for Uni. Funds	Uni/ind. collaboration	Gov. expend. on tert. education	Graduates—tertiary education	No. of patents	Scient. &technical articles	Intellect. property receipts	Trade exports	Efficiency	Uni. rank
Austria	0.6000	0.3687	0.2047	0.0500	0.0500	0.0981	0.0500	0.0050	0.4127	0.5723	0.0050	0.0050	**0.703**	**31**
Belgium	0.0500	0.0733	0.2353	0.0500	0.0500	0.3860	0.0500	0.0050	0.3337	0.5167	0.0050	0.1397	1.118	20
Canada	0.4240	0.1487	0.0500	0.0500	0.1731	0.2474	0.0500	0.0050	0.1578	0.7806	0.0517	0.0050	0.875	25
Chile	0.0500	0.1597	0.0500	0.0500	0.0500	0.0500	0.0500	0.9800	0.0050	0.0050	0.0050	0.0050	2.175	8
Czech Rep-	0.0500	0.1152	0.0500	0.0500	0.0500	0.1611	0.2219	0.0050	0.0050	0.8704	0.0050	0.1146	1.432	12
Denmark	0.6000	0.3102	0.0500	0.0666	0.0787	0.0500	0.0500	0.0050	0.3282	0.5464	0.0633	0.0571	0.830	26
Estonia	0.3169	0.6000	0.0500	0.0500	0.0000	0.6000	0.3352	0.1361	0.1063	0.6733	0.0050	0.0794	0.512	33
Finland	0.0500	0.2547	0.2137	0.0500	0.0500	0.0500	0.0500	0.0050	0.9800	0.0050	0.0050	0.0050	1.392	14
France	0.0500	0.0500	0.1192	0.1608	0.0500	0.2827	0.0500	0.0780	0.0050	0.4337	0.0994	0.3839	1.311	16
Germany	0.1489	0.0500	0.0500	0.0500	0.0500	0.5363	0.0500	0.0050	0.0050	0.0050	0.0050	0.9800	1.069	23
Greece	0.0500	0.0500	0.0573	0.0500	0.0500	0.3544	0.0500	0.0050	0.0050	0.9800	0.0050	0.0050	1.511	10
Hungary	0.0500	0.3088	0.0500	0.0500	0.0878	0.1073	0.0500	0.0779	0.0050	0.3642	0.5479	0.0050	1.420	13
Iceland	0.0503	0.0993	0.0500	0.0500	0.0500	0.0500	0.0500	0.0050	0.0050	0.0050	0.9800	0.0050	2.502	4
Ireland	0.0500	0.1929	0.0000	0.1148	0.0500	0.3299	0.0841	0.0050	0.0587	0.2637	0.0050	0.6677	1.217	18
Italy	0.0500	0.0500	0.0500	0.1384	0.0500	0.2614	0.0500	0.0050	0.0050	0.9114	0.0736	0.0050	1.539	9
Japan	0.0500	0.0500	0.0500	0.1034	0.0500	0.0500	0.0500	0.0050	0.9132	0.0050	0.0718	0.0050	2.479	6
Korea	0.0500	0.0500	0.0500	0.0500	0.0500	0.0500	0.1006	0.0050	0.0050	0.0050	0.0050	0.9800	2.496	5
Latvia	0.2085	0.6000	0.0000	0.6000	0.0500	0.6000	0.0500	0.1050	0.1590	0.7058	0.0253	0.0050	0.474	34
Lithuania	0.5480	0.0500	0.0500	0.0500	0.0500	0.0500	0.5414	0.3810	0.0050	0.5472	0.0273	0.0395	0.747	29
Luxembourg	0.0500	0.0500	0.0500	0.0917	0.0500	0.0500	0.0500	0.0050	0.0050	0.0050	0.9800	0.0050	2.553	3
Mexico	0.0500	0.0500	0.0500	0.0838	0.0500	0.3485	0.0500	0.9534	0.0050	0.0050	0.0050	0.0316	1.465	11
Netherlands	0.3062	0.0512	0.0500	0.1230	0.2323	0.6000	0.0500	0.0291	0.2184	0.5040	0.0000	0.2486	0.708	30
New Zealand	0.5833	0.0500	0.0500	0.0500	0.0500	0.0500	0.0782	0.0050	0.2810	0.6821	0.0268	0.0050	1.097	22
Norway	0.5802	0.2810	0.0646	0.0628	0.0500	0.2108	0.0500	0.0050	0.2566	0.7284	0.0050	0.0050	0.770	28
Poland	0.0500	0.0500	0.0822	0.0753	0.0508	0.0666	0.0500	0.1280	0.0050	0.8570	0.0050	0.0050	2.354	7
Portugal	0.2095	0.0500	0.0500	0.1383	0.0500	0.1639	0.0987	0.0050	0.0050	0.9800	0.0050	0.0050	1.315	15
Slovak Rep	0.1103	0.0500	0.0500	0.6000	0.0500	0.0500	0.0500	0.2823	0.0050	0.7027	0.0050	0.0050	1.041	24
Slovenia	0.0500	0.0500	0.0500	0.0500	0.0847	0.0500	0.0500	0.0050	0.2613	0.2344	0.4943	0.0050	2.599	2
Spain	0.3365	0.0927	0.0500	0.0500	0.0505	0.6000	0.0500	0.0865	0.0845	0.7067	0.0000	0.1223	0.813	27
Sweden	0.0500	0.0500	0.0500	0.0566	0.0500	0.5921	0.0500	0.0050	0.0445	0.1576	0.7879	0.0050	1.113	21
Switzerland	0.0500	0.0500	0.0500	0.0500	0.0500	0.4746	0.0500	0.0050	0.0459	0.0313	0.5679	0.3499	1.291	17
Turkey	0.3778	0.0987	0.0500	0.0500	0.2746	0.6000	0.0500	0.2180	0.0736	0.6866	0.0000	0.0218	0.666	32
UK	0.2667	0.0500	0.0500	0.0500	0.1078	0.2900	0.0500	0.0050	0.0050	0.6486	0.0836	0.2578	1.157	19
USA	0.0500	0.0500	0.0500	0.0500	0.0500	0.0500	0.0500	0.0050	0.0050	0.0050	0.9800	0.0050	2.857	1
